# Non-Invasive Respiratory Assessment in Duchenne Muscular Dystrophy: From Clinical Research to Outcome Measures

**DOI:** 10.3390/life11090947

**Published:** 2021-09-10

**Authors:** Francesca Pennati, Antonella LoMauro, Maria Grazia D’Angelo, Andrea Aliverti

**Affiliations:** 1 Dipartimento di Elettronica, Informazione e Bioingegneria, Politecnico di Milano, 20133 Milano, Italy; antonella.lomauro@polimi.it (A.L.); andrea.aliverti@polimi.it (A.A.); 2 Scientific Institute IRCCS E. Medea, 23842 Bosisio Parini, Italy; grazia.dangelo@lanostrafamiglia.it

**Keywords:** DMD, respiratory function, respiratory muscles, pulmonary function testing, imaging

## Abstract

Ventilatory failure, due to the progressive wasting of respiratory muscles, is the main cause of death in patients with Duchenne muscular dystrophy (DMD). Reliable measures of lung function and respiratory muscle action are important to monitor disease progression, to identify early signs of ventilatory insufficiency and to plan individual respiratory management. Moreover, the current development of novel gene-modifying and pharmacological therapies highlighted the urgent need of respiratory outcomes to quantify the effects of these therapies. Pulmonary function tests represent the standard of care for lung function evaluation in DMD, but provide a global evaluation of respiratory involvement, which results from the interaction between different respiratory muscles. Currently, research studies have focused on finding novel outcome measures able to describe the behavior of individual respiratory muscles. This review overviews the measures currently identified in clinical research to follow the progressive respiratory decline in patients with DMD, from a global assessment to an individual structure–function muscle characterization. We aim to discuss their strengths and limitations, in relation to their current development and suitability as outcome measures for use in a clinical setting and as in upcoming drug trials in DMD.

## 1. Introduction

Duchenne muscular dystrophy (DMD) is a X-linked myopathy caused by the deficiency of dystrophin protein. Without dystrophin, muscle fibers are gradually replaced by connective and adipose tissue and progressive weakness and atrophy of locomotor and respiratory muscles arise [1]. The progressive failure of respiratory muscles leads to respiratory complications including hypoventilation, nocturnal desaturation, and inefficient cough [2,3,4,5]. It is therefore of vital importance to identify relevant and reliable respiratory outcome measures to detect early signs of ventilatory insufficiency, to monitor disease progression and to plan individual respiratory management [2,5]. Indeed, the support of respiratory devices, namely cough assisted device and mechanical ventilation, becomes crucial [2,5]. Moreover, the currently development of novel gene-modifying and pharmacological therapies [6,7,8,9,10] highlighted the urgent need of novel respiratory outcomes to evaluate these therapies.

Spirometry is recommended by current guidelines for routine lung function evaluation in DMD [11,12], since forced vital capacity (FVC) has prognostic value for survival [13] and it is a useful guidance for treatment [11]. Nevertheless, spirometry requires a high level of patient cooperation, as well as repeated efforts that might strain older patients. In addition, spirometry provides only a global evaluation of lung restriction, without distinguishing between impairment of the respiratory pump and/or lung/chest wall stiffness. It is therefore important to distinguish the specific involvement of individual respiratory muscles by examining their structural and functional characteristics. This review aims to overview the measures which have been identified in clinical research to follow the progressive respiratory involvement in patients with DMD, from a global assessment of respiratory function to the structure–function characterization of the single muscle.

## 2. The Act of Breathing

Breathing occurs because respiratory muscles work together to generate pleural pressure swings that drive expansion or contraction of the chest wall and, thus, pump air (flow) in and out of the lungs [14,15]. The equation of motion of the respiratory system governs the relationship between the time course of these variables (pressure, flow and volume), and it depends on the mechanical properties of the system (resistance and dynamic compliance). When a disease affects one of these actors, this interferes with the functional capacity of the others. A vicious circle onset that may potentially trigger a cascade of events that facilitates the development of respiratory insufficiency occurs. Lower values of lung or chest wall (as for DMD) compliance, as well as higher levels of respiratory resistance (as for DMD in case of mucus plugging in the peripheral airways) represent an important mechanical overload for the respiratory muscles [16]. Affected respiratory muscles (as for DMD) may result in hypoventilation, rapid and shallow breathing pattern, and asynchronous breathing movement, resulting in increased cost of breathing and hypercapnia [17]. Indeed, the ultimate goal of breathing is to guarantee the proper gas exchange and to avoid respiratory failure (i.e., inadequate gas exchange by the respiratory system when the arterial oxygen, carbon dioxide or both cannot be kept at normal levels). For this reason, in addition to volume (of the lung and of the chest wall), flow and pressure, both the oxygen and the carbon dioxide concentrations are important variables of interest to be measured.

The respiratory muscles are the engine that generates the pressure gradient that moves the air in and out. According to the direction of the flow, they can be inspiratory (the diaphragm, the external intercostal muscle, the sternocleidomastoid and the scalene muscles) or expiratory (abdominal muscles and internal intercostal muscles). With their contraction, both the diaphragm and the abdominal muscles increase abdominal pressure contributing to change the volume of the abdomen, although with opposite effects. The diaphragm contracts during inspiration, making the abdomen expand, while abdominal muscles contract during active expiration, shrinking the abdomen. Rib cage muscles act on the ribs through the pump and the bucket handle movements. All ribs upon inhalation will externally rotate and elevate, therefore expanding the thorax. The opposite occurs during exhalation. Measuring thoraco-abdominal volumes might be highly informative as well. In the last decade, structural and functional characteristics of the respiratory muscles can be directly detailed by different imaging techniques, like ultrasound (US) and magnetic resonance imaging (MRI) [18,19,20,21].

All the non-invasive techniques that assess these parameters of interest are described in the following section.

## 3. Non-Invasive Assessment of Respiratory Function

### 3.1. Pulmonary Function Tests

#### 3.1.1. Lung Volume

The lung volumes of clinical interest are either capacities (i.e., absolute volumes) or variation of volume. The latter are assessed through spirometry, being part of the routine pulmonary function testing, used to assess the general respiratory health. Spirometry measures the maximal inhalation and exhalation volumes of air as a function of time via a spirometer. Traditional parameters include forced expiratory volume in 1 s (FEV1, the volume of air exhaled forcefully in the first second after a maximal inspiration to total lung capacity) and forced vital capacity (FVC, the total volume of air forcefully exhaled after a maximal inspiration to total lung capacity). In contrast to FVC, slow vital capacity (SVC) measures the maximum amount of air that can be exhaled after a maximum inspiration, without requiring a forceful maneuver. Thus, it is ideal in patients who may have difficulty in performing forceful maneuvers [22]. The measure of absolute lung volumes, including total lung capacity (TLC), functional residual capacity (FRC), and residual volume (RV) can be measured with either nitrogen washout technique or body plethysmography. These techniques have the advantages of being non-invasive, widely available and with normative values obtained from previous population study data [23,24,25]. Nevertheless, spirometry requires a high level of patient cooperation, usually scarce in pediatric patients. In older patients, suboptimal results may occur when loss of bulbar or facial muscle strength prevents the formation of a tight lip-seal around the mouthpiece [22,26] as well as when the respiratory muscles become too weak. The mouthpiece related problems occur also when measuring absolute volumes with washout techniques, while wheelchair bound patients can meet great difficulties in entering a body plethysmograph [22,27]. On average, these measures can be reliably performed from 6 years of age [22].

#### 3.1.2. Pressure

Maximum static pressures measured at the mouth represent a simple assessment of global respiratory muscle strength [28]. Maximal inspiratory pressure (MIP) is the maximal pressure generated during maximal inspiratory effort starting at residual volume, against a closed shutter sustained for 1 s, whereas maximal expiratory pressure (MEP) is the maximal expiratory effort measured starting TLC [28]. To standardize the maneuver and to maximize the potential contractility, MIP and MEP are measured from a starting volume where the inspiratory and expiratory muscles, respectively, are maximally stretched. Additionally, MIP and MEP are volitional maneuvers, therefore relying on patients’ collaboration, and they require full effort that can be difficult to be reliably performed in these patients [28]. As an alternative to MIP, the sniff nasal inspiratory pressure (SNIP) is considered a more natural maneuver to assess inspiratory muscle function. SNIP is recorded by a pressure transducer connected to a catheter placed in the nostril, with the subject instructed to sniff quickly and deeply from FRC. The main limitation of SNIP is the underestimation of inspiratory muscle strength in case of nasal obstruction by adenoids or nasal polyps, or in patients with severe respiratory muscle weakness [29]. In studies of diaphragm contraction, sniffs were shown to approximate phrenic stimulation [30] and it has been shown a strong activation of the diaphragm and of the scalene muscles during maximal sniffs [31,32]. SNIP is therefore commonly considered an index reflecting diaphragm and global inspiratory muscle strength [33,34]. However, it was recently pointed out that SNIP is not sensitive to the group of inspiratory muscles recruited [35].

#### 3.1.3. Flow

Flow of interest can be measured during forced vital capacity, but also during cough. Peak expiratory flow (PEF) is the maximal value reached during the force exhalation and it is a useful measure to assess respiratory function, in particular the degree of obstruction in the airways, but it also depends on the strength of the respiratory muscles. Peak cough flow (PCF) is the peak flow generated during a cough maneuver, after complete inhale, measured by a pneumotachograph or a peak flow meter connected to a facemask or a mouthpiece. PCF has been used to assess airway clearance capability and expiratory muscle function, and a threshold of 270 L/min has been adopted below which airway clearance is inadequate and assisted airway clearance is needed [4].

#### 3.1.4. Ventilation Inhomogeneity

The lung clearance index (LCI), derived from the multiple breath washout (MBW) technique, is a measure of overall lung ventilation inhomogeneity, calculated from the relative ventilation required to clear a tracer gas from the lung. LCI can be measured at any age in tidal breathing, without forced breathing maneuvers. Nevertheless, clinical measure of LCI is not routinely performed as time-consuming protocols are required, including at least three MBW runs with tracer gas washout until 1/40th of its starting concentration [36].

#### 3.1.5. Transcutaneous Gas Monitoring

Transcutaneous measurement of carbon dioxide pressure (Pco2) and pulse oximetry are non-invasive techniques that offer continuous monitoring over several hours by means of a transcutaneous sensor, which is placed on a membrane on the skin, commonly a digit, an ear, or the forehead. The gold standard to diagnose sleep-disordered breathing is polysomnogram. However, due to the limited access to polysomnograms, oximetry and capnography are also used in clinical practice. The overnight recording of Pco2 and oxy-hemoglobin saturation (SpO2) can determine whether a patient meets the criteria for nocturnal hypoventilation. However, it is not able to differentiate among types of sleep-disordered breathing. Hypercapnia is defined as Pco2 > 50 mmHg for 25% or more of the recording time [37], even if some studies consider this definition too restrictive [38].

### 3.2. Ventilatory Pattern

During quiet breathing, the ventilatory pattern can be recorded breath-by-breath, by measuring the temporal trend of flow, using flowmeters, or volume.

The lung volume is the integration of the flow signal. The chest wall volume can be directly measured in a precise way through optoelectronic plethysmography (OEP) or estimated by respiratory inductive plethysmography (RIP) or by structured-light plethysmography (SLP). OEP is a motion capture system that accurately measures the thoraco-abdominal contribution to tidal volume, detecting the 3D displacement of a large set of reflective markers placed on the thoraco-abdominal surface of the patient via infrared TV cameras, using dedicated geometrical models [39,40]. RIP measures variations in cross-sectional areas of two bands with incorporated inductive coils, placed around the ribcage (under the armpits) and around the abdomen (at the level of the umbilicus). The inductive current changes induced by the length changes of the coils during the expansion/contraction of the ribcage and abdomen is used to continuously monitor changes in their diameters and calculate volume changes and breathing rate [41,42]. SLP is a non-contact, self-calibrating method of assessing chest wall volume, by projecting a light in a grid/checkerboard pattern onto the patient’s chest wall [43,44]. Starting from volume trace over time, tidal volume (VT) and breathing frequency (fR) are derived. From these measures, minute ventilation (VE = VT*fR) and rapid and shallow breathing index (RSBi = fR/VT) can be obtained. As measured during quiet breathing, with no requirement for patient cooperation, these measures can be performed at any age and in different postures: standing, seated, and supine.

In case of hypoxemia and hypercapnia, the body attempts to recover by increasing both tidal volume and respiratory rate. Breathing frequency is therefore an important sensitive indicator of increasing respiratory difficulty [45]. Recently, continuous measurement of respiratory rate can be achieved by wearable devices, which derive a respiratory-related signal by detecting the motion of the thoraco-abdominal surface via inductive, resistive or capacitive sensors [46,47,48,49]. Inertial sensors mounted on the external surface of the chest or abdomen represent an emerging approach to measure chest wall breathing motions, deriving breathing signal, and related parameters. Mono- or tri-axial accelerometers systems have demonstrated their feasibility to measure breathing frequency in different positions and dynamic conditions (walking, light exercises), distinguishing among different respiratory patterns [50,51,52,53,54]. This approach is highly promising, as it allows long recordings with no need to change the habits of the patients.

### 3.3. Thoraco-Abdominal Breathing Motion

The chest wall is anatomically composed of two compartments: the ribcage and the abdomen. The tidal volume, therefore, is the sum of the volume variation of these compartments. OEP, RIP and SLP automatically provide thoraco-abdominal volume variation over time, as well. OEP is based on the anatomical definition of the two compartments since markers are placed according to anatomical points. Ribcage volume is therefore obtained as the volume enclosed by the clavicles and the lower costal margin; abdominal volume as the volume enclosed by the lower costal margin and the iliac crests [40]. SLP identified the two compartments according to geometric consideration based on square shape [55]. Finally, RIP estimates the two compartmental traces starting from the cross-sectional area of the two 2.5 cm wide, lightweight elastic and adhesive bands, therefore suffering from potential underestimation of the whole compartment [41,42].

### 3.4. Imaging

#### 3.4.1. Ultrasound (US)

US imaging allows to non-invasively study the diaphragm at rest. Using an anterior subcostal view, the diaphragm is identified as a single thick echogenic line and the excursion of the diaphragm as shown in M mode, can be measured, during tidal breathing, deep inspiration or sniff maneuvers. Using a high frequency linear probe positioned perpendicularly to the chest wall in the intercostal space around the eighth rib, the diaphragm is outlined by two clear bright parallel lines, corresponding to the pleural and peritoneal membranes. Diaphragm thickness is estimated as the distance between the two lines. During inspiration, the diaphragm thickens because of muscle contraction. The thickening fraction can be therefore calculated as the difference between end-inspiratory thickness and end-expiratory thickness, and expressed as percentage of end-expiratory thickness [18,19,20,56,57]. A major concern in respiratory muscle US is the large variability in the methodology, including variations in transducer placement, subject position and respiratory maneuver, leading to poor comparability between studies [58].

#### 3.4.2. Magnetic Resonance Imaging (MRI)

On MR images, the lungs can be directly imaged in 2D or 3D using both a static and dynamic acquisition. Using static MRI, lungs volumes and maximum vertical and antero-posterior lung diameters, as well as their changes from expiration to inspiration can be measured [59,60,61]. By combining the expiratory and the inspiratory scan, diaphragmatic movement area (DMA, i.e., the area bordered by diaphragm edges after subtracting the expiratory from the inspiratory scan) and chest wall movement area (CWMA, i.e., the area calculated subtracting DMA from inspiration) have also been measured [61]. Reconstructing the 3D shape of the diaphragm as the bottom surfaces of the segmented left and right lungs, the inspiratory–expiratory diaphragm excursion can be investigated in 3D [59,62]. Using dynamic MRI, these parameters can be measured in each frame of the cine-MRI dataset, during free- and deep-breathing conditions, and their temporal profile can be obtained [62,63,64,65]. Free-breathing acquisitions have the advantage of being performable also in patients who have difficulties holding their breath. Nevertheless, breath-hold acquisitions provide both higher spatial resolution and shorter scan durations for patients who cannot stay in the scanner for a long time or cannot perform multiple deep-breath cycles.

MRI can be also used to directly image pathological muscle changes using multi-sequence imaging protocols, including T1-weighted and T2-weighted spin echo sequences, as well as fat-suppressed T2-weighted sequences (T2WFS). To quantify muscle involvement, scoring scales, from normal appearance to complete fatty degeneration, have been proposed [66,67]. Nevertheless, scoring systems suffer from inter-subject variability and insensitivity to subtle changes over time. For an objective quantification of fat fraction, chemical shift-encoded MRI (Dixon imaging) has been introduced [68]. Dixon MRI techniques quantify the individual contributions of fat and water in each voxel of tissue, encoding the chemical shift difference between water and fat by acquiring images with slightly different echo times [69]. Schematically, a first acquisition is performed at an echo time when protons of fat and water are in phase and a second acquisition is performed at an echo time when water and fat are out of phase. The addition or subtraction of the resultant in-phase or out-of-phase images produces fat and water images, in which the intensity of individual pixels is proportional to fat or water concentration in that voxel. Dixon techniques can be implemented in both gradient echo and spin-echo sequences, with some technical adaptation, using either multi-repetition or multi-echo methods [70]. Recently, it has been demonstrated that the amount of fat tissue in skeletal muscle biopsy correlated significantly with the fat fraction derived from the Dixon sequence [71]. As an alternative quantitative approach, T2 relaxation time mapping (T2 maps) can be used to evaluate muscle disease involvement in neuromuscular disorders, reflecting both fatty infiltration and inflammation/edema from muscle damage.

MRI provide a direct measure of both respiratory muscles structure and function. However, contraindications to MRI such as metal implants, invasive ventilation, and claustrophobia make it impossible to scan certain patients.

## 4. Respiratory Assessment in Duchenne Muscular Dystrophy

### 4.1. Respiratory System

#### 4.1.1. Lung Volume

With the disease progression in DMD, the respiratory muscles become weaker and taking a deep breath becomes harder for patients. Inspiratory reserve volume consequently reduces resulting in a lower total lung capacity (TLC). Moreover, exhalation is limited by the progressively acquired decreased chest wall compliance, producing a higher residual volume (RV). Forced vital capacity (FVC) is the most widely used outcome measure to assess respiratory function. Being the difference between TLC and RV, FVC is a global measure of both inspiratory and expiratory muscles’ function as well as chest wall stiffness. In DMD, FVC peaks between ages 10 and 14 years, whereas FVC%linearly declines, with variable rates depending on the age range of the patients included in the study [72,73,74,75]. The most robust studies on the natural history in DMD have reported that FVC% starts around the restrictive value of 80%, it remains stable until 10–11 years of age and it further declines at a constant rate of 5–6%/year [76,77]. LoMauro et al. investigated the volume components which lead to FVC decline, reporting significantly lowered inspiratory capacity and expiratory reserve volume, after the ages of 6 and 8 years, respectively [77]. Additionally, a reduction of TLC and FRC compared to predicted starting from 7.7 and 9.3 years old, respectively, and an increased RV after the age of 11.3 years was reported [77].

On dynamic MRI, lung cross sectional area was reported smaller in patients than in controls at both tidal and maximal inspiration and expiration [63,64,65]. These results were also confirmed when lung cross sectional area was normalized to height, to account for shorter stature resulting from long-term steroid treatment [63].

#### 4.1.2. Flow

In the absence of obstructive lung disease, PEF has been demonstrated highly reliable in DMD (coefficient of variation about 7%), and therefore highly valuable to assess expiratory muscle strength [78]. Yearly rate of PEF decline has been reported to be about 8.9% in patients aged 10–18 years [78]. Nevertheless, we note that PEF measures the peak instantaneous flow during a forced expiratory maneuver after a full inspiratory breath and therefore can be impacted both by inspiratory and expiratory muscle strength together. Based on the measurement of peak inspiratory flow during a forced vital capacity maneuver and during a tidal breathing, inspiratory flow reserve has been proposed as a measure of inspiratory function loss in DMD [79]. In a study evaluating the effect of idebenone, both peak inspiratory flow and inspiratory flow reserve have been used as outcome measures [80].

### 4.2. Ventilation

#### 4.2.1. Ventilatory Pattern

In patients with DMD, minute ventilation progressively declines in supine position, significantly after the age of 18 years [77]. Patients develop hypoventilation, due to the progressive decrease of tidal volume, but preserved respiratory rate [77,81]. In a pilot study using an inertial measurement unit (IMU-based device) in patients with DMD, Cesareo et al. proposed the overtime measurement of breathing frequency during the autonomous daily use of the system, to predict respiratory dysfunction, reporting higher breathing frequencies associated with worse respiratory function [82].

#### 4.2.2. Thoraco-Abdominal Breathing Motion

The abdominal contribution to tidal volume during awake resting inspiration progressively decreases with age in supine position starting at the age of 15 years [77,81], but also in seated position [81], while the thorax expands more [77,81]. In addition, the supine abdominal tidal volume turns to be a predictor of nocturnal hypoexemia [83]. Indeed, patients experiencing nocturnal oxygen desaturation were characterized by significantly lower abdominal expansion compared not only to healthy peers, but also to DMD peers with normal oxygenation while sleeping [81,83]. Finally, DMD patients with inefficient cough were characterized by reduced thoraco-abdominal volumes during the inspiration preceding cough but also by reduced abdominal contribution to tidal volume during quiet breathing, that is also a predictor of inefficient cough [84].

#### 4.2.3. Ventilation Inhomogeneity

In a group of 45 patients with DMD aged 5–25 years old, it has been demonstrated an increased ventilation inhomogeneity associated with the progressive decline in lung volume, with the retention of airways secretion and/or altered geometry suggested as possible causes [85].

### 4.3. Gas Exchange

#### Transcutaneous Gas Monitoring

Sleep related hypoventilation in DMD, which precedes daytime hypoventilation, is due to both decreased ventilatory drive and respiratory muscle weakness and often obstructive sleep apnea, related to upper airways weakness, coexists [86,87,88,89]. The development of nocturnal hypoventilation in DMD has been demonstrated also in case of normal spirometry [86,87]. Hypoventilation during sleep has been well described in patients with moderate-to-severe restrictive pulmonary defects and advanced muscle weakness [89,90], but it has been more recently reported also in younger patients, aged 11 years old [87].

### 4.4. Respiratory Muscles

#### 4.4.1. Pressure

In a group of 64 DMD patients aged 10–18 years, Meier et al. reported high coefficients of variability for MIP (18%) and MEP (15%), irrespective of age, reflecting the patients’ difficulties of reliably reproducing these measures [78]. MIP and MEP have demonstrated an earlier impairment than other PFTs, with values below the lower limit of normal also at about 6–7 years of age [76], with a yearly decline of about 4–7% [91,92]. The earlier and more severe decrease of MEP than MIP has suggested the relative sparing of the diaphragm in relation to accessory respiratory muscles [75,76]. The reduced reliability and the earlier age at which these values reach floor levels limit their usefulness as clinical trial endpoints. In a group of 51 children with Duchenne and Becker muscular dystrophy, aged 5–20 years old, SNIP measurement was found highly reproducible (coefficient of variability 8%) [93]. Nève et al. also reported that SNIP decreases earlier than vital capacity and peak expiratory flow in subjects with DMD, demonstrating its value as an early marker of inspiratory strength decline [93,94]. In another study of 55 DMD patients aged from 6 to 19 years old, SNIP values decline by about 6% predicted per year [92].

#### 4.4.2. Diaphragm Excursion

In patients, excursion amplitude during quiet breathing and sniff maneuvers on US are reduced, as well as excursion velocity during sniff maneuvers [95]. On MRI, the maximal excursion decreases with age and early signs of diaphragm weakness have been reported in patients younger than 15 years old, with reduced excursion both in the dome area [59,65] and near the posterior costal margin [59]. Paradoxical diaphragm descent rather than elevation during maximal expiration has been observed in some patients with advanced disease [59,63].

#### 4.4.3. Diaphragm Thickness and Thickening Ratio

On US, diaphragm thickness in patients under 14 years old increases at FRC compared to healthy controls and reduces at TLC suggesting a decrease in diaphragm contractility, due to diaphragmatic pseudo-hypertrophy [56,57]. In older boys, the progressive atrophy of the diaphragm leads to decreasing thickness at both FRC and TLC and decreasing thickening ratio [57]. In a group of patients aged 21–31 years old, decreasing right and left diaphragmatic thickening ratio have also been reported when measured during a sniff maneuver, with an inverse relationship with age [95].

#### 4.4.4. Fatty Infiltration

On MRI, in a group of 36 individuals with DMD aged 7–19 years old, evidence of respiratory muscle pathology has been reported in early stages of the disease, when patients are still ambulant and highly functional [63]. In particular, the internal oblique shows the earliest signs of involvement, followed by the external obliques and rectus abdominis, with median fat fraction in the overall group, respectively, equal to 49%, 41% and 33% [63]. In a group of 26 DMD patients aged 8–32 years old, fat fraction of the diaphragm was found about 30% and 70%, respectively, below and above 15 years old [59], reaching 50% at around 17 years old. Therefore, diaphragm impairment results slower if compared to other muscle groups that reaches 50% fat fraction before 15 years old, including expiratory muscles [63], paraspinal muscles [59], quadriceps, hamstrings, and adductors [96]. Interestingly, in the age range 7–19 years old, the intercostals appear to have less fatty infiltration on visual assessment [63]. MRI measurements of the T2 relaxation time of the sternocleidomastoid muscles revealed altered tissue composition in patients with no respiratory symptoms, and this alteration appeared to precede myocardial alteration [97].

## 5. Respiratory Outcomes to Link Structural and Functional Respiratory Impairment

**Figure 1:**
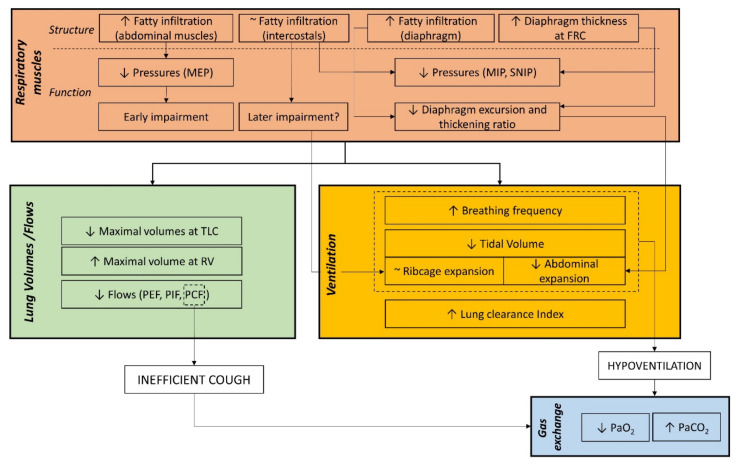
DMD progressively activates a cascade of events that starts from a structural change of the muscles and ends in their impaired function ultimately resulting in ventilatory failure.

DMD progressively activates a cascade of events that starts from a structural change of the muscles and ends in their impaired function ultimately resulting in ventilatory failure as shown in Figure 1. The deficiency of dystrophin protein of the respiratory muscles makes muscle fibers be gradually replaced by connective and adipose tissue with different timing in the three groups of respiratory muscles. Abdominal muscles seem to be the earliest involved, when patients are still ambulant and highly functional [63], with 50% fat fraction reached before 15 years old. The diaphragm, the most important respiratory muscle, experiences a slower impairment, with significantly higher fat fraction and atrophy than healthy subjects after 15 years old [57,59]. Intercostal muscles appear to have limited fatty infiltration, even in patients with nearly complete fatty replacement of other muscles surrounding the ribcage [63].

Such structural damage induced by the dystrophy results in an impaired function, because of the loss of contracting units, with reduced diaphragm excursion and ventro-dorsal lung expansion [59,63,64]. Additionally, in severe advanced stage, paradoxical motion of the diaphragm has been reported during maximal expirations [59,63]. It can be hypothesized that this paradoxical descent rather than elevation may be the result of diaphragm co-contraction during maximal expiratory efforts [98], together with its slower impairment if compared to other expiratory muscles [59,63]. Additionally, the reduced maximal pressures indicate that all the respiratory muscles experience fatigue. Muscle fatigue is defined as the decrease in maximal force or power produced in response to contractile activity [99]. As for respiratory muscles the force of contraction is translated into pressure development, the reduced MIP and SNIP indicate that the overall inspiratory muscles develop progressive weakness. Similarly, decreased values of MEP indicate that weakness arises in the overall expiratory muscles. The earlier and more severe decline of MEP than MIP is coherent with the earlier impairment, with increased fat fraction, of expiratory muscles compared to the diaphragm [59,63]. Due to the nature of the measurements, it was not possible to specifically address information of the single group of respiratory muscles, which would require the measurements of esophageal and gastric pressures. However, these measurements require the use of two trans-esophageal catheters that may result invasive in DMD patients, because of the bulbar involvement, and this goes beyond the purpose of this review [34]. The reduced pressure development, being the gradient that moves the air between the ambient and the alveoli, results in reduced flow generation and lung expansions. The reduced capacity of generating flow leads to ineffective cough and to retained airways secretion, that may explain the increased lung clearance index [85]. To verify this hypothesis, it would be of interest to couple the measurement of LCI to ventilation imaging, such as hyperpolarized-gas MRI or inspiratory–expiratory ventilation maps [100,101]. As all the respiratory muscles are involved, a restrictive pattern is present at both maximal inspiratory and expiratory capacities, resulting in a progressively reduced vital capacity. However, maximal pressures, flows and volumes start to be significantly lower since early childhood, presumably because of the maximal efforts they required from the dystrophic respiratory muscles. For this reason, they are not sensitive enough to predict early sign of nocturnal hypoventilation or ventilatory failure during the evolution of the disease, as it is difficult to identify points of inflection that might predict aggravation of the respiratory function and represent milestones of clinical interventions. At rest, the ventilatory parameters start to become significantly impaired in late teens with the reduced tidal volume as the leading cause of hypoventilation, after the age of 18 years. The restriction at rest was entirely located in the abdomen (and therefore a reduced action of the diaphragm that expands the abdomen), not compensated by the ribcage muscles [81].

## 6. Respiratory Outcomes for the Clinical Management of Patients with DMD

Regular monitoring of pulmonary function and gas exchange is crucial in patients with DMD, to recognize early symptoms of sleep-disordered breathing, daytime hypoventilation, and inefficient cough and swallowing ability and to timely implement the appropriate treatments. Guidelines for the respiratory management of DMD indicates lung-volume recruitment techniques, manual and mechanically assisted cough and non-invasive ventilation support as critical therapies [1,11]. These interventions have been linked with prolonged survival [102,103,104].

Lung volume recruitment technique is undertaken by using a self-inflating manual ventilation bag or mechanical insufflation/exsufflation once or twice daily and is indicating when FVC is lower than 60% of predicted. Lung volume recruitment has demonstrated to improve respiratory system compliance [105] and to significantly decrease the rate of decline of FVC %pred [106,107]. Other physiotherapy interventions, aiming at training respiratory muscles, have been suggested to be beneficial in DMD, but their effect is still controversial, as they could accelerate fatigue of weakened muscles and cause muscle damage due to overwork of the muscles [108]. Moreover, the effects of respiratory muscle training in patients with DMD vary, with some studies suggesting positive results and others showing minimal or insignificant changes in respiratory muscle strength and endurance [12]. Expiratory muscle training has shown beneficial effects on expiratory muscle strength and augmented MIP [109,110], whereas inspiratory muscle training allows diaphragmatic training in terms of strength and endurance [108]. It has been hypothesized that there may be benefits to carefully prescribe inspiratory and expiratory muscle training in DMD [111], but further research is required. In this framework, quantitative imaging of individual respiratory muscles may help to evaluate the efficacy of different exercise regimens, distinguishing between safe and damaging activities.

Ineffective cough and consequent retained secretion is the main cause of episodes of pneumonia and acute respiratory failure, secondary to benign upper-respiratory infection. Studies have identified a peak cough flow (PCF) of 160 L/min as threshold value for starting cough-augmentation therapy [12]. However, PCF above 160 L/min does not guarantee adequate airway clearance, as respiratory muscle function can deteriorate during respiratory infections. Therefore, a PCF of 270 L/min has been suggested by the ATS Consensus to identify patients who would benefit from starting cough-augmentation therapy [12]. The measurement of MEP was also proposed to assess cough strength, with MEP > 60 cmH_2_O indicative of effective cough [112]. Additionally, pulse oximetry measurement in addition to PCF can help to identify patients at risk of developing acute respiratory failure, for whom an increased frequency of assisted coughing is indicated [113]. More recently, in a study on 36 DMD patients, LoMauro et al. identified the abdominal contribution to tidal volume during spontaneous breathing as a sensitive index able to discriminate between efficient and inefficient cough [84]. When assisted coughing is recommended it should be used once or twice per day as maintenance therapy and should be increased in case of respiratory infections [114]. A great variety of cough augmentation techniques can be considered to support airway clearance, categorized into assisted inspiration, assisted expiration, or a combination of assisted inspiration and expiration. Techniques that target the inspiratory phase of cough aim to maximally inflate the lungs, and can be achieved by glossopharyngeal breathing or air stacking [115]. Compared to glossopharyngeal breathing, air stacking was demonstrated to better improve the ability to increase lung volume and to permit greater assisted PCF [116]. The expiratory phase can be spontaneous if the subject is still able to generate sufficient intrathoracic pressures or assisted, manually by abdominal compression or mechanically forced by a cough assisted device [115]. Studies suggest that the combination of inspiratory support with assisted expiratory cough is more efficacious in augmenting PCF than a single technique [117,118].

Nocturnal ventilation is indicated when signs of insufficient pulmonary function are present during sleep. Initial recommendations for initiation of non-invasive ventilation were based on daytime hypoventilation [12], but gradually it has been introduced earlier in the progression of sleep disordered breathing [11,119]. The most recent guidelines indicated initiation of NIV based on transcutaneous pCO_2_ (>50 mmHg for 2% of sleep time or increased by 10 mmHg from awake baseline) and SpO_2_ (≤88% for 2% of sleep time or for at least 5 min uninterrupted). Several studies investigated the sensitivity of spirometry parameters to respiratory muscle weakness, which could be useful as daytime predictors of sleep breathing related disorders and indicators for establishing nocturnal non-invasive ventilation [5,13,89,120], but a consensus is still missing. A set of indexes based on different combinations of breathing pattern and spirometric parameters have also been proposed [121,122]. More recently, in patients with DMD who present either no or mild nocturnal oxygen desaturation and who do not receive non-invasive mechanical ventilation, abdominal contribution to tidal volume and inspiratory capacity demonstrated sensitivity to mild desaturation during sleep [83]. In the later stages of the disease, patients develop daytime dyspnea and hypercapnia, and nocturnally assisted ventilation is extended into daytime and, ultimately, into 24-h-per-day ventilation. Assisted daytime ventilation is initiated if the SpO_2_ < 95%, pCO2 > 45 mmHg, or symptoms of awake dyspnea are present [11]. The traditional approach to the last stage of the disease is to introduce tracheostomy ventilation [123,124], but the need for and timing of tracheostomy is debated. The 24-h-per-day NIV, such as mouthpiece ventilation, is entirely possible and represents a safe non-invasive alternative to tracheostomy, allowing the patient a greater ability for natural speech and swallowing [102,125,126,127]. Some centers initiate assisted ventilation noninvasively and then transition patients to tracheostomy as their pulmonary function declines. Tracheostomy ventilation becomes necessary in case of severe aspiration risk and severe respiratory infection.

Swallowing deficit should be also evaluated in patients with advanced disease, as it represents the principal cause for decreased oral intake and it is closely associated with bulbar muscle function. A significant interaction has been reported between respiratory muscle strength, evaluated by measuring MIP and MEP, and both nutritional parameters [128] and the presence of eating disorders [129]. In addition, malnutrition may affect diaphragm thickness and function [130]. Moreover, the altered respiratory pattern in DMD affects the swallowing process, with frequent occurrences of inspiratory effort immediately after swallowing which may increase the risk of aspiration [131].

## 7. Respiratory Outcomes for Clinical Trials

There is currently no definitive cure for DMD, but the field of DMD therapy is advancing very quickly. The choice of primary and secondary outcome measures to evaluate the efficacy of clinical trials is crucial. Outcome measures should be quantitative, sensitive to longitudinal changes, safe and repeatable. Moreover, outcomes strongly related with quality of life, such as initiation of non-invasive ventilation or number of recoveries/year due to respiratory infections, render the findings of the studies clinically meaningful.

The current standard of care for DMD consists of corticosteroids [11], which has demonstrated to preserve muscle strength and to delay loss of ambulation, need for ventilatory support and development of cardiomyopathy [132]. An overall improved lung function, including FVC, FEV1, PEF, MIP and MEP, has been reported in patients who are current recipients of steroid therapy, across the age groups 10–12 and 13–15 years old [76,77,133]. However, long-term corticosteroid treatment leads to comorbidities, including short stature, obesity, and osteoporosis.

Idebenone, an antioxidant that improves mitochondrial energy production, demonstrated the reduced loss of respiratory function in patients with DMD [74]. Results from a randomized, placebo-controlled phase 2 trial in 21 DMD patients aged 8–16 years (DELPHI trial) provided initial evidence of the beneficial effects of idebenone in slowing the loss of respiratory function, with improved PEF %pred in patients treated with idebenone compared to patients receiving placebo, over a 12-month treatment period [80,134]. In a randomized, placebo-controlled phase 3 trial (DELOS trial), the efficacy of idebenone on respiratory function outcomes has been specifically investigated in 64 DMD patients aged 10–18 years, showing a reduction in the loss of respiratory function over a 1-year period, based on percent predicted FEV1, FVC and PEF [74,78]. Additionally, a positive impact of idebenone on inspiratory muscle function has been reported, based on maximum inspiratory flow and inspiratory flow reserve [135]. The reduced decline in percent predicted FVC and PEF was maintained for several years, as reported in a retrospective cohort study (SYROS) from 18 patients [136]. Currently, the effect of idebenone specifically on respiratory measures, selected as primary and secondary outcomes, are under investigation in a phase III trial (SIDEROS) in DMD patients taking glucocorticoids (NCT02814019, NCT03603288) [137].

New therapies to increase dystrophin production in small genetic subsets of DMD have recently been approved. Ataluren in EU and eteplirsen in US are the first mutation-specific therapies to gain regulatory approval and others are under development or near regulatory review. The effect of ataluren on lung function has been investigated in a prospective study including four patients and no statistically significant improvement/stabilization was detected [138]. More recently, a trend toward delayed worsening of pulmonary function for ataluren-treated patients has been suggested [139], but the younger age and shortened duration of follow up of the treated subjects compared with controls did not permit firm conclusions. Respiratory variables have been recently investigated in trials on ataluren as secondary (NCT01009294, NCT02090959) and primary (NCT03552874) outcomes in addition to other clinical and functional measures [137]. The long-term effect of eteplirsen has been described on numerous pharmacodynamic and functional outcomes [140]. The effect of eteplirsen on lung function has demonstrated its beneficial effect in slowing respiratory decline with a significant reduction in the rate of decline of percent predicted FVC, MEP and MIP [141,142]. Significant attenuation of annual percent predicted FVC over 96 weeks, has been recently reported for the phase 3, multicenter trial (PROMOVI trial) including 79 patients [143]. Currently, respiratory measures have also been included in another clinical trial on eteplirsen as secondary outcomes (NCT03992430) [137].

Currently, FEV1, FVC and PEF are the respiratory measures included as primary and secondary outcomes in clinical trials (NCT03879304, NCT04371666, NCT02500381, NCT03340675). In addition, MIP, MEP and SNIP have been included as outcomes in trials investigating the effects of different physiotherapy programs, to determine respiratory muscle strength (NCT03879304, NCT03963453). Interestingly, respiratory muscle MRI is currently included as secondary outcome in a study on the potential of noninvasive MRI to monitor disease progression and to serve as an outcome measure for clinical trials in muscular dystrophies (NCT01484678) [137].

## 8. Conclusions

Different outcomes have been demonstrated useful to monitor respiratory disease progression, to be used to plan individual respiratory management and to evaluate novel therapies.

The more traditional measures, such as maximal pressures, flows and volumes have an almost linear decline starting from early childhood and therefore it is difficult to identify specific time points that represent milestones for respiratory clinical management. As effort-dependent measures, they require patient cooperation, coordination and motivation and therefore cannot be performed in early childhood and late adolescence. Moreover, they represent measures of overall respiratory impairment and their decline results from the interaction between respiratory muscles, lungs, airways resistance and chest wall compliance. Nevertheless, these measures are highly available and reproducible across clinical centers and studies on the natural progression of respiratory function decline are available [76,77], making these measures the preferential outcomes for clinical management and clinical trials.

Over these measures, the evaluation of breathing pattern and thoraco-abdominal volumes are effort-independent and well suited for the evaluation of respiratory function starting from early childhood. Moreover, studies on the natural progression of these outcomes over time are available. They have demonstrated sensitivity to early ventilatory insufficiency, and they represent highly valuable outcomes to guide clinical management, providing milestones to start cough assistance and nocturnal ventilation [77,83,84]. Nevertheless, these measures require specialized equipment, trained personnel and therefore are not easily applicable across different centers and in clinical trials.

Imaging techniques provide specific measures of structural and functional impairment of respiratory muscles. Ultrasound, as an effort independent technique, may be particularly interesting for use in clinics and for experimental trials, but the lack of standardization, the high intra- and inter-operator variability impede its widespread clinical use. Other ultrasound techniques such as strain imaging or elastography of the diaphragm, respectively sensitive to muscle contractility and stiffness [144,145,146], may provide potentially interesting outcomes. Additionally, ultrasound of extra-diaphragmatic respiratory muscles, such as parasternal intercostals and abdominal muscles, has not been applied in neuromuscular disease [147,148,149]. Still, strict protocols and personnel training should be defined for the widespread clinical use of ultrasound techniques. On the contrary, MRI of the respiratory muscles is more dependent on patient’s cooperation, in relation to performance of breathing maneuvers and lying in the scanner, but it is more reproducible across clinical centers. Using MRI protocols based on standard routine sequences, readily available on clinical imaging units, measures of muscle involvement in DMD are highly reproducible across multiple vendor systems and radiofrequency coil configurations [150].

Respiratory function has been only recently included as primary outcome in clinical trials and this is particularly important, as respiratory failure is among the main causes of death in DMD. An increased number of studies have proposed novel non-invasive measures for respiratory assessment and research for standardizing protocols across different centers is ongoing. The development of measures aimed to specifically understand the separate involvement of the different actors of breathing, may provide a tool not only for evaluating clinical trials, but also to guide clinical decisions from the early stage of the disease.

## Data Availability

Not applicable.
